# Identification of Proteins Associated with the Early Restoration of Insulin Sensitivity After Biliopancreatic Diversion

**DOI:** 10.1210/clinem/dgaa558

**Published:** 2020-08-24

**Authors:** Cecilia Karlsson, Kristina Wallenius, Anna Walentinsson, Peter J Greasley, Tasso Miliotis, Mårten Hammar, Amerigo Iaconelli, Sofia Tapani, Marco Raffaelli, Geltrude Mingrone, Björn Carlsson

**Affiliations:** 1 Late-stage Development, Cardiovascular, Renal and Metabolism, BioPharmaceuticals R&D, AstraZeneca, Gothenburg, Mölndal, Sweden; 2 Department of Molecular and Clinical Medicine, Institute of Medicine, Sahlgrenska Academy, University of Gothenburg, Gothenburg, Sweden; 3 Research and Early Development, Cardiovascular, Renal and Metabolism, BioPharmaceuticals R&D, AstraZeneca, Gothenburg, Mölndal, Sweden; 4 Fondazione Policlinico Universitario A. Gemelli IRCCS, Rome, Italy; 5 Early Biometrics and Statistical Innovation, BioPharmaceuticals R&D, AstraZeneca, Gothenburg, Mölndal, Sweden; 6 Università Cattolica del Sacro Cuore, Rome, Italy; 7 Department of Diabetes, King’s College London, London, United Kingdom

**Keywords:** afamin, apolipoprotein A-II, apolipoprotein A-IV, biliopancreatic diversion, insulin resistance, proteomic analysis

## Abstract

**Context:**

Insulin resistance (IR) is a risk factor for type 2 diabetes, diabetic kidney disease, cardiovascular disease and nonalcoholic steatohepatitis. Biliopancreatic diversion (BPD) is the most effective form of bariatric surgery for improving insulin sensitivity.

**Objective:**

To identify plasma proteins correlating with the early restoration of insulin sensitivity after BPD.

**Design:**

Prospective single-center study including 20 insulin-resistant men with morbid obesity scheduled for BPD. Patient characteristics and blood samples were repeatedly collected from baseline up to 4 weeks postsurgery. IR was assessed by homeostatic model assessment for insulin resistance (HOMA-IR), Matsuda Index, and by studying metabolic profiles during meal tolerance tests. Unbiased proteomic analysis was performed to identify plasma proteins altered by BPD. Detailed plasma profiles were made on a selected set of proteins by targeted multiple reaction monitoring mass spectrometry (MRM/MS). Changes in plasma proteome were evaluated in relation to metabolic and inflammatory changes.

**Results:**

BPD resulted in improved insulin sensitivity and reduced body weight. Proteomic analysis identified 29 proteins that changed following BPD. Changes in plasma levels of afamin, apolipoprotein A-IV (ApoA4), and apolipoprotein A-II (ApoA2) correlated significantly with changes in IR.

**Conclusion:**

Circulating levels of afamin, ApoA4, and ApoA2 were associated with and may contribute to the rapid improvement in insulin sensitivity after BPD.

It is well established that bariatric surgery has profound effects on body weight, insulin resistance (IR) and obesity-associated comorbidities (1). Different bariatric surgery techniques have overlapping, but also distinct changes in key biological pathways (2). Biliopancreatic diversion (BPD) is the bariatric surgery technique with most pronounced effect on IR (3). In fact, following BPD, insulin sensitivity per kg body weight exceeds that of normal weight subjects (3).

IR is a risk factor for development of a variety of diseases including type 2 diabetes (T2D), diabetic kidney disease (DKD), cardiovascular disease (CVD), and nonalcoholic steatohepatitis (NASH) (4-7). Indeed, patients with greatest IR before bariatric surgery, measured as elevated fasting insulin levels or homeostatic model assessment for insulin resistance (HOMA-IR), are the ones with most pronounced cardiovascular and renal outcome benefits after surgery (8-10). Additionally, there are reports describing the beneficial effect of bariatric surgery on resolution of NASH (11, 12).

The present study focused on the early changes in IR, body weight, inflammation, and plasma proteins in response to BPD with the aim to identify plasma proteins that may be responsible for the early restoration of insulin sensitivity after BPD.

## Material and Methods

### Study design

**Figure 1.**
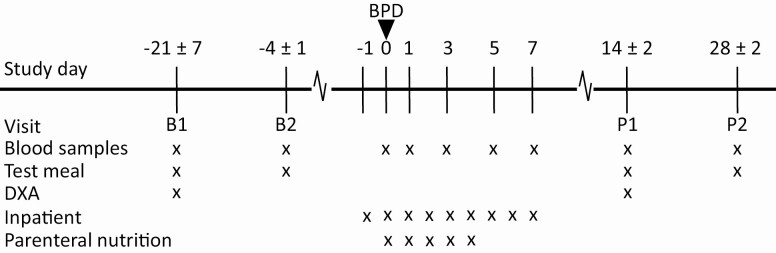
Outline of study protocol. Timing of the visits in relation to Day zero when the biliopancreatic diversion (BPD) surgery was performed. Blood sampling and study procedures were conducted at visits illustrated with an x.

Twenty severely IR men with morbid obesity scheduled for BPD were included in this single-center, observational study performed at the Fondazione Policlinico Gemelli IRCCS, Catholic University, Rome, Italy. Figure 1 shows the timing of visits and procedures in relation to Day zero, when the BPD surgery was performed. Subjects were examined and blood samples collected on 2 occasions before surgery, baseline-1 (B1, −21 ± 7 days) and baseline-2 (B2, −4 ± 1 day); on the day of BPD surgery (Day 0); every second day after surgery during the first week (immediate postsurgery); as well as postsurgery-1 (P1; 14 ± 2 days after surgery) and postsurgery-2 (P2; 28 ± 2 days after surgery). Fasting samples were obtained in the morning after an overnight fast (12 hours). Immediate postsurgery fasting samples were collected in the morning after a 12-hour rest from the parenteral nutrition which was administered during the first 4 days after surgery to avoid energy restriction. From Day 5 the subjects started diet re-aliment and on Day 8 they were on a free diet. Nutrient stimulation samples were obtained at B1, B2, P1, and P2 after intake of a test meal. Dual-energy x-ray absorptiometry (DXA) analysis of body composition was performed on B1 and P1.

### Study population

The inclusion and exclusion criteria were set to create a sufficiently homogenous population. **Inclusion criteria:** Men affected by morbid obesity (body mass index [BMI] >40 kg/m^2^) who were eligible for bariatric surgery and accepted to undergo BPD; confirmed IR by HOMA-IR; fasting serum insulin level >8.6 mU/L; age 25 to 55 years; weight stable for at least 6 months before the study (± 5 kg within the previous 6 months); and stable medications if present. **Exclusion criteria:** Patients not eligible for BPD; incapacity to give a valid informed consent or unwilling to give the consent; patients eligible for BPD, but with T2D; significant illness within the 2 weeks preceding surgery (as judged by the physician), obvious infection, major cardiovascular, gastrointestinal, or respiratory disease or any hormonal disorders; medication affecting lipid metabolism within 3 months of the study; history of drug addiction and/or alcohol use; suspected or confirmed poor compliance; exercise > 3 times a week; blood donation within 12 weeks preceding screening visit.

The study was performed in accordance with the ethical principles in the Declaration of Helsinki, Good Clinical Practice, and applicable regulatory requirements and was approved by the Ethical Committee of the Catholic University in Rome. All participants provided written informed consent to participate in the study.

### Treatments

BPD was performed by open surgery and included removal of the gallbladder. The procedure is an approximately 60% distal gastric bypass with stapled closure of the duodenal stump as previously described (13). Subjects received a parenteral nutrition regimen (Periven Fresenius Kabi Italia, 7113 kJ/day [1700 kcal/day]) during the first 4 days after surgery. Four times/day 2 g cephalosporin (Pfizer Italia Srl) was administered from Day −1 to Day 3.

### Meal tolerance test

A meal tolerance test (MTT) was performed at 4 different occasions, B1, B2, P1, and P2. The meal consisted of 15 g Weetabix, 10 g skimmed milk, 250 mL pineapple juice, 50 g white chicken meat, 60 g whole meal bread, and 10 g polyunsaturated margarine. The total energy was 2015 kJ (482 kcal) and carbohydrate accounted for 75 g (14). The meal was eaten within 10 minutes. At B1 and P1 full plasma profiles (−30, −1, 10, 20, 30, 40, 50, 60, 75, 90, 120, 150, 180, 210, and 240 minutes) were collected whereas at B2 and P2 only fasting (t = −20 minutes) and fed (t = 90 minutes) samples were obtained.

### Sample collection

Blood samples for biochemical analyses were collected in EDTA-evacuated tubes with the addition of a dipeptidyl peptidase IV inhibitor (LC0014; Linco, St. Charles, MO, USA) and in standard lithium-heparin tubes. Blood samples for proteomic analysis were collected in BD P100 vacuum tubes and handled according to the manufacturer’s instructions (BD Biosciences, NJ, USA). Plasma samples were kept cold and immediately separated by centrifugation at 4 °C. All samples were stored at −80 °C until analyses.

### Analyses

Plasma glucose was measured by the glucose oxidase method (Beckman, Fullerton, CA, USA). Insulin was assayed by microparticle enzyme immunoassay (Abbott, Pasadena, CA, USA). Nonesterified fatty acid (NEFA), triglyceride, total cholesterol, and high-density lipoprotein were measured by enzymatic colorimetric methods at Catholic University clinical laboratories. C-reactive protein (CRP) was measured in serum by an enzyme-linked immunosorbent assay (ELISA) with a detection limit of 1 ng/mL (DRG International Inc, Springfield, NJ, USA). Radioimmunoassays were used for C-peptide (MYRIA, Technogenetics, Milan, Italy), total glucagon-like peptide-1 (GLP-1) (Linco research, St Charles, MO, USA), and gastric inhibitory polypeptide (GIP) (Peninsula Laboratories, Belmont, CA, USA).

### Anthropometrical measurements

Body weight was measured to the nearest 0.1 kg on a balance beam scale. The measurements were done in the morning before breakfast after a visit to the lavatory with subjects wearing only underwear. Height was measured to the nearest 0.5 cm using a stadiometer. Waist circumference was measured by a Gulick II Tape at the part of the trunk midway between the most caudal part of the lateral costal arch and the iliac crest with the person standing with their feet 25 to 30 cm apart. The waist circumference was measured to the nearest 0.5 cm at the end of a normal expiration. Hip circumference was measured as the maximal circumference over the buttocks. Sagittal diameter was recorded in supine position just above the anterior superior iliac spine corresponding to L4–L5.

### Calculations

BMI was calculated as weight (kg) divided by height^2^ (m^2^). Low-density lipoprotein was calculated according to Friedewald et al (15). Calculations of IR was done by HOMA-IR using the formula (fasting insulin [mU/L] × fasting glucose [mmol/L])/22.5 and Matsuda Index of Insulin Sensitivity was calculated as previously described (16). The area under the curve (AUC) during the MTT was calculated for each individual and analyte (A) by calculating the area ((A_t1_+A_t2_)/2) (t_2_-t_1_) between each sampling time point (−30, −1, 10, 20, 30, 40, 50, 60, 75, 90, 120, 150, 180, 210, and 240 minutes) and summing this across the whole sampling period.

### Dual-energy x-ray absorptiometry

Total body DXA was performed using a Lunar DPX absorptiometer (Lunar Radiation Corp., Madison, WI, USA) in slow mode, and data were analyzed using software version 3.6z. Data collected from DXA include fat mass, lean body mass, and total body bone mineral density.

### Unbiased proteomics

Nano-flow liquid chromatography coupled to tandem mass spectrometry (LC-MS/MS) analysis was performed by Proteome Sciences PLC (London, UK) on 80 plasma samples derived from the 20 subjects sampled at B1 before MTT, B1 after MTT, P2 before MTT, and P2 after MTT. Abundant proteins were depleted using the Qproteome Albumin/IgG Depletion Kit (Qiagen, #37521). Samples were digested using trypsin, labeled with TMT8plex including an internal reference from a pool of all samples, and followed by SCX fractionation and LC-MS/MS (MS3) analysis, using the EASY-nLC II NSI Orbitrap Velos (Thermo) system. Data processing and protein identification were carried out using Proteome Discoverer 1.3 (Thermo) software. An in-depth description of the unbiased proteomics workflow can be found in the Supplemental Materials, Text S1 (17).

### Targeted proteomics

A targeted multiple reaction monitoring mass spectrometry (MRM/MS) assay was developed and validated for absolute quantification of 11 selected proteins. The proteins were selected from the set of proteins that were significantly altered in response to surgery and based on feasibility to analyze the proteins by MRM/MS. A customized MRM kit including stable isotope-labeled peptide standards (SIS) was purchased from MRM Proteomics Inc. (BC, Canada). The samples analyzed were derived from 20 subjects sampled at 9 different time-points (eg, all fasting blood samples collected as shown in Fig. 1). Detailed information of the targeted proteomic workflow is outlined in the Supplemental Materials, Text S2 (17).

### Statistical analyses

#### Patient characteristics and biochemical analysis.

Data are reported as group means ± standard deviation and in the figures displayed with 95% confidence interval (CI). Normality assumptions were checked by means of Q-Q plots, histograms, and box-plots. In the case of single pairwise comparisons, Student *t-*test was used and *P* < 0.05 was considered statistically significant. Pearson’s correlation coefficients have been calculated to investigate potential associations. Mixed linear models were used to analyze repeated measures data, comparing all data to B1, with fixed effects for visit sequence and with a random patient effect. The analysis compensates for unbalanced missing values on visit occasions. *P* values are corrected for multiplicity through Dunnett’s method. Body weight, recognized as a potential confounder, was tested as a covariate for changes in glucose, insulin, HOMA-IR, and CRP but as the correlations were weak, nonsignificant, and results did not change the data interpretation, we have not included this analysis. One patient with very high fasting insulin levels was found to have notable impact on measures of variability and was excluded from the further analysis. A sensitivity analysis was done showing that by excluding this patient’s data the conclusions were not changed. Selected baseline data for this patient (to ensure patient anonymity), are reported in Supplemental Materials, Table S1 (17). Statistical analyses were performed in GraphPad Prism version 7.04 and the mixed linear models were performed in R version 3.4.3.

#### Unbiased proteomic data.

To assess the effects of surgery and/or MTT on plasma protein levels, 1-way analysis of variance (ANOVA) was conducted with 4 experimental variables corresponding to the different time-points (B1, t = −20 [before MTT]; B1, t = 90 [after MTT]; P2, t = −20 [before MTT]; P2, t = 90 [after MTT]). A prerequisite for this analysis was the requirement that the proteins were present in at least 40 of 80 samples (50%) with quantitative data available in the LC-MS/MS study (leaving 289 eligible proteins). After filtering, the data matrix was normalized to their quantiles. Bonferroni method was used to adjust for multiplicity. Proteins were considered significantly changed when the adjusted *P* value was equal to or smaller than 0.05.

#### Identification of proteins correlating to insulin resistance and CRP. 

The quantified protein profiles by MRM/MS were obtained from plasma samples collected in the fasting state at 9 time-points throughout the study. The profiles were visually inspected, and subsequently analysis of the correlations of the MRM/MS profiles to IR and CRP was performed.

#### Bioinformatic analysis.

To elucidate the biological mechanisms modulated by BPD, Gene Ontology (GO) term overrepresentation analysis was performed using PANTHER (http://pantherdb.org/), using the 29 proteins regulated by BPD as test set, and all human genes in the database (N = 21 042) as reference set. GO terms, representing Biological Processes (BPs), Molecular Functions (MFs) and Cellular Components (CCs), were considered significant based on Fisher’s exact test with corrected *P* value (FDR) < 0.05.

## Results

Baseline characteristics are shown in Table 1. Patients were men with morbid obesity and fasting insulin levels >8.6 mU/L. Body fat constituted 44.0 ± 3.7% of total body weight and lean mass accounted for 50.1 ± 3.5%.

**Table 1.** Baseline Characteristics of Patients at B1 and B2. All Individuals Were Male. n = 19

	B1	B2
**Age (y)**	36.5 ± 8.0	
**Height (cm)**	180.5 ± 7.9	
**Weight (kg)**	156.1 ± 14.4	156.3 ± 14.1
**BMI (kg/m** ^**2**^)^**a**^	48.2 ± 5.1	
**Waist circumference (cm)**	145.1 ± 13.5	145.1 ± 13.3
**Hip circumference (cm)**	139.6 ± 13.6	139.6 ± 13.6
**Sagittal diameter (cm)**	61.3 ± 11.5	61.4 ± 11.5
**DXA LM (kg)**	78.1 ± 8.1	
**DXA FM (kg)**	68.6 ± 8.7	
**DXA BMC (kg)**	1.4 ± 0.1	
**HR (bpm)**	72.4 ± 6.7	69.1 ± 11.2
**Systolic BP (mmHg)**	128.4 ± 4.4	127.4 ± 5.1
**Diastolic BP (mmHg)**	79.7 ± 3.1	80.5 ± 4.4
**Glucose (mmol/L)**	4.7 ± 1.0	5.1 ± 1.0
**Insulin (mU/L)**	26.0 ± 9.5	31.0 ± 14.0
**C-peptide (ng/mL)**	5.0 ± 3.3	4.6 ± 1.0
**C-reactive protein (mg/L)**	8.8 ± 4.4	8.9 ± 4.0
**Total cholesterol (mmol/L)**	4.9 ± 1.2	
**HDL-cholesterol (mmol/L)**	1.0 ± 0.2	
**LDL-cholesterol (mmol/L)**	3.1 ± 1.0	
**Triglycerides (mmol/L)**	1.5 ± 0.6	
**HOMA-IR** ^**b**^	5.45 ± 2.45	6.97 ± 3.55
**Matsuda Index** ^**c**^	2.8 ± 1.13	

Abbreviations: BMC, bone mineral content; BMI, body mass index; BP, blood pressure; DXA, dual-energy x-ray absorptiometry; FM, fat mass; HDL, high-density lipoprotein; HOMA-IR, homeostatic model assessment for insulin resistance; HR, heart rate; LDL, low-density lipoprotein; LM, lean mass.

^a^BMI is calculated as weight (kg) divided by height^2^ (m^2^)

^b^HOMA-IR = (fasting insulin (mU/L) x fasting glucose (mmol/L))/22.5

^c^Matsuda Index of Insulin Sensitivity based on test meal (16)

**Figure 2.**
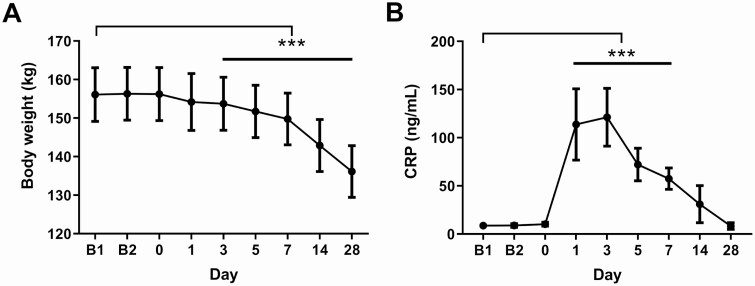
Time course of body weight and CRP changes over the study period. Body weight (A) and CRP (B) were measured repeatedly throughout the study. Data are presented as mean ± 95% CI and all comparisons are made to B1. Mixed linear models were used to analyze repeated measures data with fixed effects for visit sequence and with a random patient effect. ***P < 0.0001 except B1 vs Day 3 *P* = 0.00035. Corrected for multiplicity with Dunnet’s method. n = 15-19.

Figure 2A shows the time course of body weight change. Body weight was significantly reduced from Day 3 postsurgery. At Day 28, the subjects had lost on average 19.9 ± 2.9 kg, which corresponds to a 12.8 ± 1.9% weight reduction from baseline. Measures of waist circumference and sagittal diameter were both reduced postsurgery (Supplemental Materials, Figures S1A-B (17)). DXA analysis showed a reduction in fat mass of 3.4 ± 3.0 kg and lean body mass 7.5 ± 3.5 kg at 14 days after surgery (*P* ≤ 0.0001 for both measures). As surgery is associated with tissue injury and an inflammatory response, the magnitude of this response was assessed by measuring CRP. CRP was significantly elevated during the first 7 days after surgery (*P* < 0.0001) before returning to baseline levels at Day 28 (Fig. 2B).

**Figure 3.**
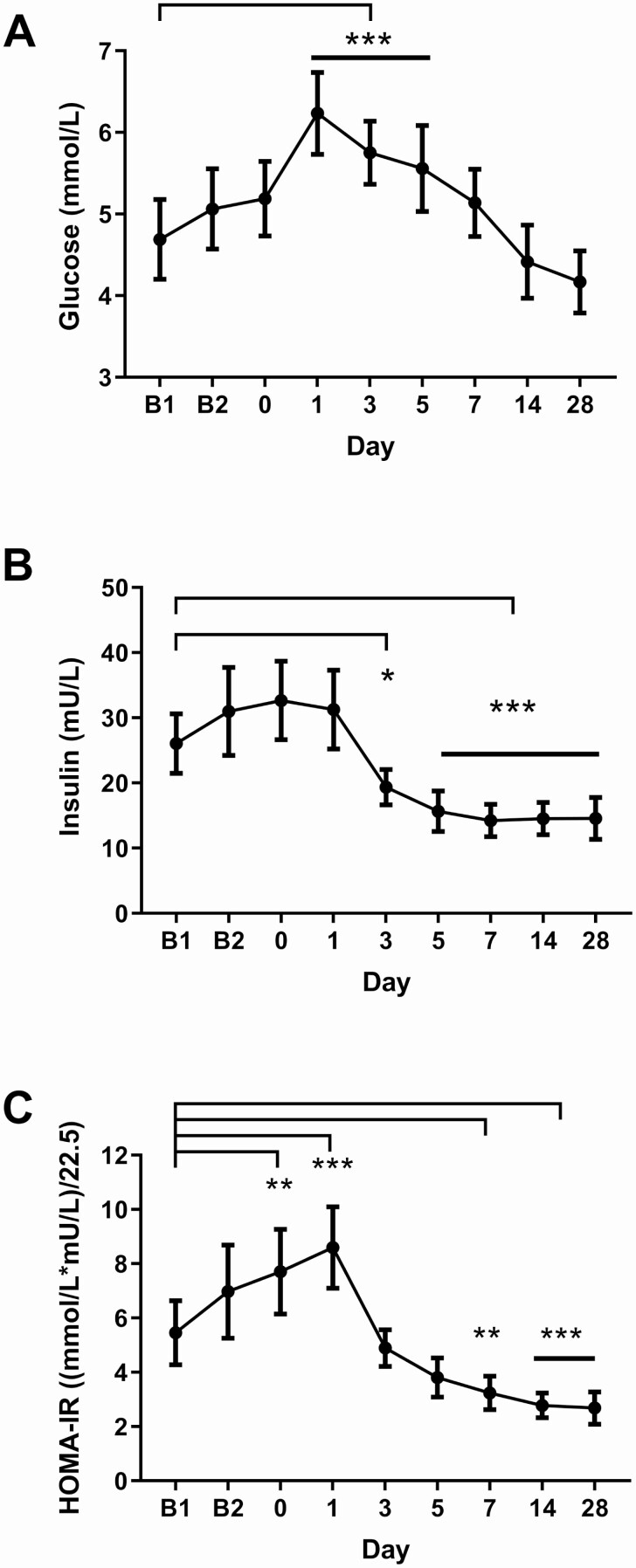
Rapid reduction in insulin resistance following BPD. Fasting plasma glucose (A), insulin (B) and HOMA-IR (C) were measured throughout the study. Data are presented as mean ± 95% CI and all comparisons are made to B1. Mixed linear models were used to analyze repeated measures data with fixed effects for visit sequence and with a random patient effect. Corrected for multiplicity with Dunnet’s method. A) ***P < 0.0001 except Day 5 vs B1 *P* = 0.000155; B) *P = 0.0455, ***P < 0.0001 except Day 5 vs B1 *P* = 0.000210; C) Day 0 vs B1 **P = 0.00621, Day 1 vs B1 ***P < 0.0001, Day 7 vs B1 **P = 0.00455, Day 14 vs B1 ***P = 0.000254, Day 28 vs B1 ***P = 0.000145. n = 15-19.

Fasting plasma glucose levels increased during the first days after surgery returning to baseline levels between Day 7 and 14 (Fig. 3A). Fasting insulin levels were reduced by Day 3 postsurgery relative to baseline (*P* = 0.0455, Fig. 3B) and continued to fall until plateauing at Day 7 postsurgery. In line with increased glucose levels at the time of surgery, HOMA-IR was increased on Days 0 and 1 before reaching a significant reduction compared to B1 from Day 7 (Fig. 3C).

**Figure 4.**
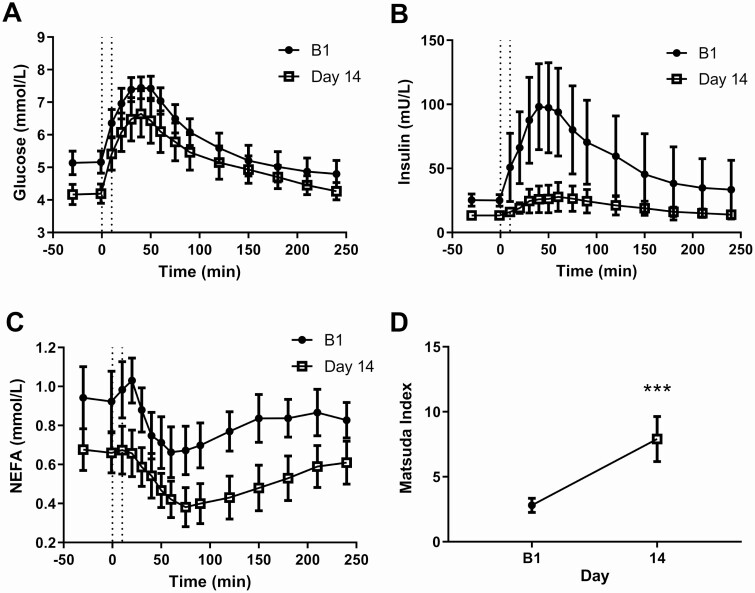
Improved metabolic control 14 days after BPD. Glucose (A), Insulin (B), NEFA (C) and Matsuda index (D) following a meal tolerance test (MTT) on B1 and Day 14 after BPD. Data are presented as mean ± 95% CI. Statistical testing was performed on the AUC and on mean fasting values collected just before the MTT (−30 and −1 minutes), see results for values (A-C). Paired *t-*test was used for Matsuda index. *** *P* < 0.0001. n = 19.

An MTT with detailed blood sampling was performed at baseline (B1) and at Day 14 postsurgery (P1). Plasma profiles for glucose, insulin, and NEFA following the MTT are shown in Fig. 4A-4C. Basal fasting glucose levels (mean of −30 and −1 minute) were elevated at baseline relative to 14 days after BPD (Glucose B1: 5.1 ± 0.7, Day 14: 4.2 ± 0.6 mmol/L, *P* < 0.0001). The AUC for the glucose excursion was reduced following BPD on Day 14 (B1: 1552 ± 193.6, Day 14: 1383 ± 240.4, *P* = 0.0002) (Fig. 4A). Fasting basal insulin levels were almost halved postsurgery (B1: 25.2 ± 9.5, Day 14: 13.3 ± 4.3 mU/L, *P* < 0.0001) and insulin AUC was reduced by 64% (B1: 14 701 ± 13 949, Day 14: 5233 ± 3000, *P* = 0.0062, Fig. 4B) following the MTT. Fasting plasma NEFA levels were reduced before the MTT at Day 14 following BPD (B1: 0.93 ± 0.32, Day 14: 0.67 ± 0.22 mmol/L, *P* = 0.0073) (Fig. 4C). In response to the MTT at B1, NEFA levels increased slightly before starting to fall, reaching a minimum value of 0.66 ± 0.27 mmol/L at 60 minutes. In comparison, on Day 14, NEFA levels started to rapidly decline following the challenge, reaching trough levels of 0.38 ± 0.21 mmol/L at 75 minutes. Matsuda index was significantly increased Day 14 compared to B1 (Fig 4D, *P* < 0.0001). Plasma levels of GLP-1 increased rapidly and peaked after 30 minutes in response to the MTT both at baseline and 14 days post-BPD (Supplemental Materials, Figure S2A (17)). Both fasting GLP-1 levels (B1: 25.6 ± 9.5, Day 14: 28.4 ± 9.5 pg/mL, *P* = 0.0009) and the AUC following the MTT were increased postsurgery (AUC B1: 7697 ± 2906 and P1: 8553 ± 2836, *P* = 0.0004). Plasma levels of GIP were not changed in response to surgery (Supplemental Materials, Figure S2B (17). There was no significant difference between fasting triglyceride at B1 and Day 14, however in response to the MTT the triglyceride AUC was significantly decreased (Supplemental Materials, Figure S2C (17), AUC B1: 531 ± 215 and P1: 434 ± 159, *P* = 0.0139).

**Figure 5.**
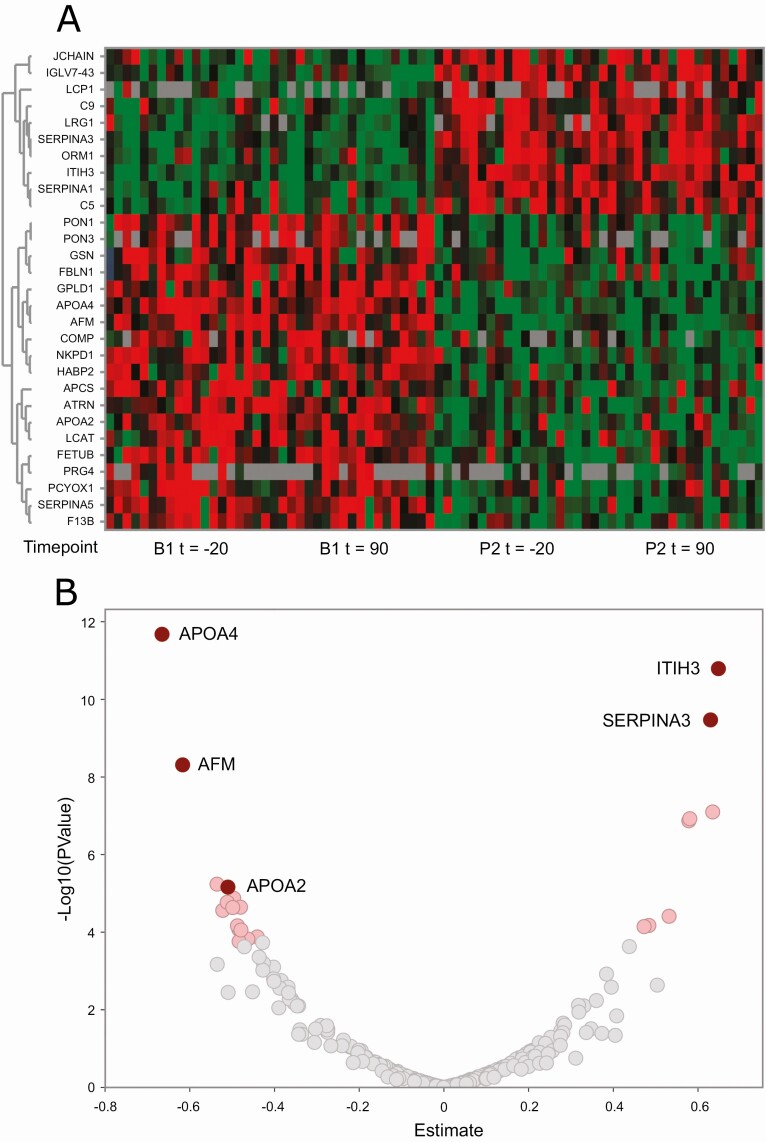
Change in protein expression following BPD. Unbiased LC-MS/MS proteomics identified 29 unique proteins that were significantly changed between baseline and 4 weeks postsurgery. Heatmap (A) visualizes that 10 proteins were upregulated and 19 were downregulated in response to surgery. The horizontal axis shows the 4 time-points when the samples were collected: fasted before MTT (t = −20), fed 90 minutes after initiation of MTT (t = 90) before and after BPD (B1 and P2, respectively) and on the vertical axis the gene symbols corresponding to the differentially regulated proteins are shown. The Volcano plot (B) shows the magnitude of change (x-axis) and statistical significance (y-axis) of the complete set of 289 assessed proteins in fasting samples only. Proteins appearing on the right and left of x = 0 are up- and downregulated, respectively, in response to BPD. Significantly changed proteins are colored in light red and selected proteins referred to in the text are labeled with gene symbols.

Unbiased proteomics was used to identify plasma protein level changes following BPD. Supplemental Materials, Figure S3 (17) describes all steps used to identify proteins correlated with HOMA-IR. ANOVA performed on the LC-MS/MS data showed 29 proteins significantly changed between B1 and P2 (Supplemental Materials, Table S2 (17)). Significantly changed proteins are presented in a clustered heat map of the normalized LC-MS/MS data (Fig. 5A; red, increased- and green, decreased levels); 10 proteins were increased while 19 proteins had reduced levels. There were no significant differences between fasting and fed states either before or after surgery. A volcano plot of the complete set of 289 proteins assessed by ANOVA shows the statistical significance versus the magnitude of change (*y*- and *x*-axis, respectively) and separates the top altered proteins based on increased (right side of 0 on the *x*-axis) or decreased (left side of 0 on the *x*-axis) amounts in response to BPD (Fig. 5B); significantly changed proteins (ie, with adjusted *P* value ≤ 0.05) are marked in light red. The plasma proteins most altered in response to BPD are inter-alpha-trypsin inhibitor heavy chain 3 (ITIH3) and alpha-1-antichymotrypsin (SERPINA3) (both upregulated), as well as apolipoprotein A-IV (ApoA4), afamin, and apolipoprotein A-II (ApoA2) (downregulated).

Functional analysis of specific Gene Ontology (GO) biological processes, molecular functions and cellular components are shown in Supplemental Materials, Table S3 (17). Tissue gene expression profiles indicated that at least half of the altered proteins are selectively expressed by the liver, including *ITIH3*, *AFM*, *APOA2*, *PON1*, *ORM1*, and *FETUB*, whereas *APOA4* is the only gene that is selectively expressed by the gastrointestinal tract (GTEx consortium, www.gtexportal.org).

**Figure 6.**
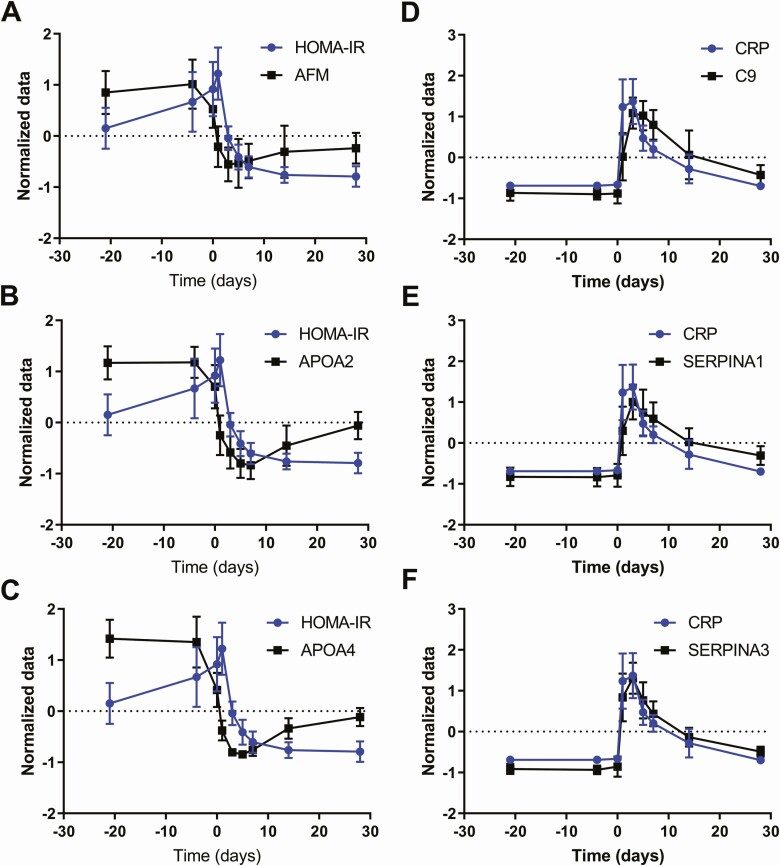
Time-resolved protein expression and correlations to HOMA-IR and CRP. Two main profiles emerged when studying the MRM/MS protein profiles in plasma samples collected in fasting state throughout the study. One following the HOMA-IR profile as shown for afamin (A), ApoA2 (B), and ApoA4 (C) and the other one following the CRP profile as shown for C9 (D), SERPINA1 (E) and SERPINA3 (F). Gene names are used for simplicity and the corresponding protein names can be found in Supplemental Materials, Table S2 (17). To facilitate graphing all values have been normalized to have mean 0 and standard deviation 1. Absolute values are found in Fig. 2B (CRP), 3C (HOMA-IR), and Supplemental Materials, Figure S4 (17) (MRM/MS data). n = 19.

Targeted MRM/MS proteomic analysis across the full time course was performed on 11 of the identified proteins (Fig. 6 and Supplemental Materials, Figure S4 (17)). The level of each protein was temporally compared to HOMA-IR and CRP, as key markers of IR and inflammation, respectively. Three proteins, afamin (*r* = 0.303, *P* = 0.000112), ApoA2 (*r* = 0.271, *P* = 0.000589) and ApoA4 (*r* = 0.355, *P* < 0.00001), significantly correlated with HOMA-IR (Fig. 6A-6C). Three other proteins, complement component C9 (C9) (*r* = 0.510, *P* < 0.00001), alpha-1-antitrypsin (SERPINA1) (*r* = 0.528, *P* < 0.00001), and SERPINA3 (*r* = 0.674, *P* < 0.00001) significantly correlated with CRP (Fig. 6D-6F). The 3 proteins correlating with HOMA-IR did not correlate with changes in body weight (BW) (afamin vs BW: *r* = 0.0851, *P* = 0.288; ApoA4 vs BW *r* = 0.103, *P* = 0.200; ApoA2 vs BW *r* = 0.144, *P* = 0.0720) in this analysis of early metabolic changes after BPD.

## Discussion

This study was designed to detect early changes in IR, body weight, inflammation, and plasma proteins following BPD to identify proteins that were associated with the early restoration of insulin sensitivity after surgery. Besides insulin, prognostic biomarkers for development of IR-related diseases (including T2D, DKD, CVD, NASH) as well as for patient selection for bariatric surgery and other insulin-sensitizing therapies are missing. Such biomarkers would facilitate early identification and treatment of patients at highest risk for disease. Furthermore, there is a need for treatments that improve insulin sensitivity (18). The identified proteins may have a role in development of IR and may be responsible for the early restoration of insulin sensitivity after BPD.

One strength of this study is the frequent sampling under standardized conditions, which allowed us to characterize the time course of events. After a transient increase in IR postsurgery, IR was reduced within days despite a significant increase in inflammatory markers, including CRP. The time-course changes in HOMA-IR, with reduced IR from Day 7 onward, were supported by results from an MTT at Day 14 showing a pronounced decrease in insulin levels with a simultaneous reduction in glucose and NEFA. The significantly reduced NEFA levels following BPD in both the fasting state and following the MTT support a direct insulin-sensitizing effect on the adipose tissue leading to reduced lipolysis.

By applying unbiased plasma proteomic analysis, followed by MRM/MS, we identified 3 proteins—afamin, ApoA4, and ApoA2—as proteins that strongly correlated with changes in IR independently of changes in body weight in the early phase after BPD. A dedicated study would be needed to understand if the rapid changes in IR are driven by a shift to a negative energy balance or by an altered intestinal nutrient sensing and signaling postsurgery (19). Afamin, ApoA4 and ApoA2 have previously been reported to be linked to the development of metabolic disease including one or more of T2D, DKD, or NASH as detailed below. IR is a risk factor for these diseases and our finding that afamin, ApoA4, and ApoA2 were changed after BPD and correlated with changes in HOMA-IR supports the idea that these proteins may be involved in the pathophysiology of IR and IR-related diseases.

Afamin is a member of the albumin gene family and is mainly expressed in the liver (20). Afamin has the strongest clinical support in the literature for its association with IR-related diseases. Noninterventional clinical studies have shown that plasma levels of afamin are strongly associated with IR, as well as prevalence and incidence of T2D (20, 21). Transgenic mice overexpressing human afamin also show increased body weight, glucose, and lipid levels, further linking afamin to the metabolic syndrome (20, 22). Treatment with glucocorticoids has been found to increase plasma levels of afamin, induce IR, and increase risk of T2D (23). Other studies have shown that urinary afamin predicts development and progression of DKD in patients with T2D and that afamin is upregulated in serum samples from patients with nonalcoholic fatty liver disease, suggesting that early identification of patients with elevated afamin levels may identify patients at high risk of IR-related diseases (24, 25). Consistent with our finding, plasma proteomic analysis after Roux-en-Y gastric bypass surgery has also shown reduced afamin levels (26).

ApoA4 is mainly produced in the small intestine, and is secreted together with chylomicron particles (27). In our study, plasma levels of ApoA4 were reduced after BPD and correlated strongly with the dramatic reduction in IR. Most available clinical data on ApoA4 are related to progression of DKD. Elevated plasma levels of ApoA4 have been shown to predict progression of chronic kidney disease in nondiabetic patients and the progression of renal impairment in T2D (28-31). Furthermore, in the Fremantle Diabetes Study, ApoA4 has been identified as a novel plasma biomarker that predicts rapid decline in renal function independently of other clinical risk factors in T2D (32). ApoA4 also seems to play a role in development of steatohepatitis (33). In contrast to afamin, where Roux-en-Y gastric bypass surgery and BPD surgery showed a consistent downregulation of afamin, there are inconsistent data in the regulation of ApoA4 with an increase after Roux-en-Y and a decrease after caloric restriction and BPD (26).

ApoA2 is mainly expressed in the liver and is a primary component of high-density lipoprotein (34). Much less is known about ApoA2 in relation to T2D and other IR-related diseases. While some reports have described ApoA2 as a candidate gene for T2D, there are conflicting data (35-37). Limited human data suggest that ApoA2 is implicated in renal amyloidosis (38).

Interestingly, the proteins correlating with changes in IR identified in our study, are all suggestive of being involved in development of kidney disease. This is in line with results from Ahlqvist et al, indicating that patients with T2D and severe IR had significantly higher risk of developing DKD than other T2D patients (39).

This study has several limitations. The inclusion of only men may impact the applicability of results across genders, while allowing creation of a sufficiently homogenous population for the key study question. HOMA-IR was selected as the outcome measure to identify correlated proteins. Although less robust than MTT, HOMA-IR provided an opportunity to generate a detailed time curve of changes in IR including also time-points directly after surgery, which had not been possible with MTT. Inclusion of additional groups, such as patients without IR and patients with T2D, as well as a longer follow-up period would have added value to this study. In addition, this study is relatively small, and findings should be replicated in independent studies.

In summary, this study has identified changes in plasma levels of afamin, ApoA4, and ApoA2 as significantly correlating with the early changes in IR in response to BPD surgery. Further studies are needed to evaluate the potential role of afamin, ApoA4, and ApoA2 as predictive biomarkers for IR-related diseases such as T2D, DKD, CVD, and NASH.

## Data Availability

Selected materials are presented in the data repositories listed in References. AstraZeneca Group of Companies is dedicated to protecting the privacy of all individuals whose data we collect, store, and/or analyze. Our first obligation is to honor the contracts with our patients, only allowing access and use as agreed in the Informed Consent Forms. As the data we publish are historical in nature, re-consent of all patients in publication datasets to allow broad access and reuse is not practical. Due to the GDPR regulations in Europe and the potential risk of re-identification of the subjects, we cannot make the individual patient data, or data derived from patient samples, available for broad open, public, use. AstraZeneca continues to support data sharing and transparency in order to enable scientific breakthroughs that improve the lives of our patients. The company has established a Data Request Portal through which Researchers can request access to de-identified clinical data (https://astrazenecagroup-dt.pharmacm.com//DT/Home/Index/). Clinical data requested through the Portal may become available after review of the patient consent forms, scientific merit of the proposal, and signature of a data sharing/collaboration agreement. This mechanism allows controlled, risk-managed accessibility of the data and at the same time safeguards patients’ confidentiality. AstraZeneca remains committed to publishing strong scientific research aimed at increasing the quality of patients’ lives of patients globally.

## Abbreviations

ANOVA: analysis of variance
AUC: area under the curve
ApoA2: apolipoprotein A-II
ApoA4: apolipoprotein A-IV
BMI: body mass index
BPD: biliopancreatic diversion
CRP: C-reactive protein
CVD: cardiovascular disease
DKD: diabetic kidney disease
DXA: dual-energy x-ray absorptiometry
GIP: gastric inhibitory polypeptide
GLP-1: glucagon-like peptide-1
HOMA-IR: homeostatic model assessment for insulin resistance
IR: insulin resistance
LC-MS/MS: liquid chromatography–tandem mass spectrometry
MRM/MS: multiple reaction monitoring mass spectrometry
MTT: meal tolerance test
NASH: nonalcoholic steatohepatitis
NEFA: nonesterified fatty acid
T2D: type 2 diabetes

## Additional Information

***Disclosure Summary:*** A.I., and M.R. have nothing to declare. C.K., K.W., A.W., P.J.G., T.M., M.H., S.T., and B.C. are employed by AstraZeneca. C.K., K.W., A.W., P.J.G., T.M., M.H., S.T., and B.C. own shares in AstraZeneca. G.M. is on the Board of Novo Nordisk, Johnson & Johnson, Fractyl Inc, and Keyron Ltd.
